# Recent Status on Lactate Monitoring in Sweat Using Biosensors: Can This Approach Be an Alternative to Blood Detection?

**DOI:** 10.3390/bios15010003

**Published:** 2024-12-24

**Authors:** Leonardo Messina, Maria Teresa Giardi

**Affiliations:** 1 Microsis srl, Via degli Olmetti, 8a, 00060 Formello, Italy; mt.giardi@biosensor.it; 2 Institute of Cristallography, CNR Area of Research of Rome, 00010 Rome, Italy

**Keywords:** lactate detection, wearable biosensors, blood/sweat correlation, electrochemical biosensors

## Abstract

Recent studies have shown that lactate is a molecule that plays an indispensable role in various physiological cellular processes, such as energy metabolism and signal transductions related to immune and inflammatory processes. For these reasons, interest in its detection using biosensors for non-invasive analyses of sweat during sports activity and in clinical reasons assessments has increased. In this minireview, an in-depth study was carried out on biosensors that exploited using electrochemical methods and innovative nanomaterials for lactate detection in sweat. This detection of lactate by biosensors in the sweat method seems to be feasible and highly desirable. From this commentary analysis, we can conclude that the correlation between lactate concentrations in sweat and blood is not yet clear, and studies are needed to clarify some key issues essential for the future application of this technology.

## 1. Introduction and Literature Search Methods

**Figure 1:**
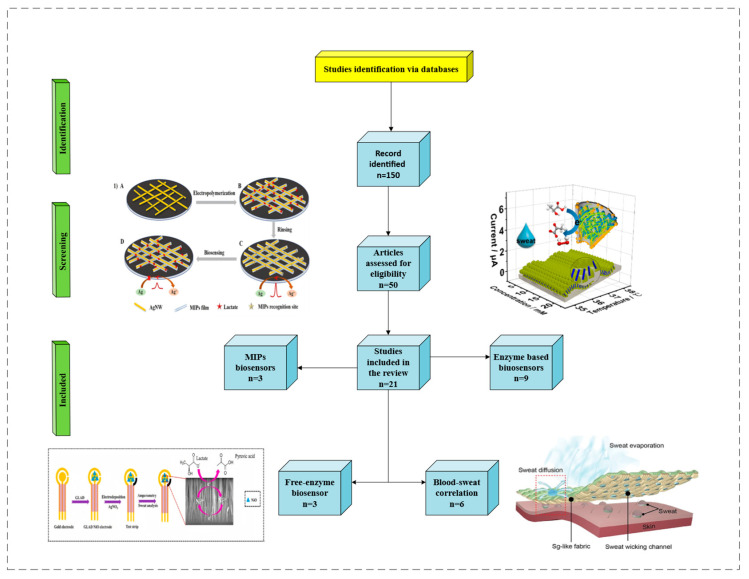
Flowchart of study selection for this minireview. Images reprinted with permission from [10,11,12,13]. Copyright © 2024 American Chemical Society; copyright 2024 Elsevier; copyright 2020 Elsevier; copyright © 1999–2024 John Wiley & Sons, Inc., or related companies.

Recently, the possibility of non-invasive and effective methodologies for biological studies has markedly increased; these methods play a prominent role in scientific research, especially in biosensor applications. Measuring sweat components is beneficial for disease diagnosis, health care, and exercise management, and it is expected to play a crucial role in various fields, including point-of-care testing [1,2]. Lactate is an important molecule present in the biological fluids of our bodies as the core of metabolic regulation in vivo. Our bodies produce it through glycolysis in anaerobic conditions. Its presence in abnormal concentrations can be a symptom of a pathology or a specific psychophysical state in an individual. The accumulation of lactate in the body is more dangerous than other molecular fuels, and an increase in serum lactate can lead to lactic acidosis [3]. Its determination is important to be able to evaluate the presence of disorders, such as shock or respiratory and heart attacks [4]. Lactate regulates the breakdown of fat and sugar for energy in athletes. However, it is also associated with muscular fatigue, and it is a major limitation in athletic performance. Lactate concentrations vary depending on the biological fluid; in blood, under normal conditions, its concentration ranges from 0.6 to 2.0 mM, while in sweat, it ranges from 4 to 25 mM [5]. Under pathological conditions, such as pressure-induced ischemia, or during intense physical activity, it can increase to 70 mM [6]. Lactate detection using sweat necessitates the development of a non-invasive and rapid methodology that differs from those already present in the market. In fact, this metabolite is usually studied via blood analysis using invasive methods. Scientific research in this field has focused on obtaining a biosensor capable of evaluating lactate concentrations in sweat in real time. Recently, due to the possibility of detecting lactate in sweat, many researchers have sought to analyze the main characteristics and features of this methodology through reviews. For example, Yang et al. [7] investigated the impact of lactate in sports, focusing on the description of electrochemical biosensors, all based exclusively on the use of enzymes. The remarkable study by Pérez and Orozco [8], on the other hand, focused on the anatomical description of the sweat glands, which are involved in lactate metabolism, and on the biochemical processes that occur during sweating. Shen et al. [9] described, in detail, the entire range of biosensors that can be applied for the detection of sweat lactate, discussing self-powered semiconductors, as well as optical and electrochemical biosensors. Compared to the above studies, this minireview exclusively focuses on a detailed analysis of recent research on electrochemical biosensors, based on both enzymatic and non-enzymatic processes, ending with the application of MIPs with an emphasis on new nanomaterials. For a complete analysis of biosensors used for lactate detection, a targeted bibliographic search was carried out, and the findings of this in-depth study reveal the extent to which the challenges in lactate detection are not addressed, as most studies are limited to the technical results obtained without discussing the possibility of applying electrochemical biosensors for the detection of this analyte in sweat. For this reason, after a careful analysis of articles, we performed a targeted screening of only the most recent and innovative articles on the use of nanomaterials for electrochemical biosensors. A flowchart of the literature search and identification of relevant articles for this minireview is depicted in Figure 1. Of the 21 studies included, 15 articles were categorized and reviewed (Table 1). For the analysis of the correlation between lactate concentration in sweat and blood in Section 3, the remaining six articles are considered. Furthermore, a market analysis was carried out to gain insight into the state of the art of these biosensor technologies (Table 2).

## 2. Electrochemical Biosensors and Nanomaterials for Lactate Analysis in Sweat

Many types of biosensors can be used for lactate detection, including optical, spectroscopic, and electrochemical biosensors. Electrochemical devices may involve different techniques, such as the use of enzymes, which are capable of providing high selectivity and high sensitivity under external environmental conditions. Inspired by the metal architecture in nanomaterials, scientists have developed artificial biocatalysts or functionalized nanozymes that are more stable and specific and ensure effective electrochemical response with little dependence on detection conditions, such as pH and temperature. Nanozymes involve the formation of a non-enzymatic substrate that undergoes a redox reaction with the analyte of interest. For example, using nickel-based nanozymes, it is possible to develop NiOOH, a well-known and efficient electrocatalyst for lactate oxidation [14]. Another type of widely used electrochemical biosensor involves MIPs, which are polymers capable of selectively recognizing the analyte of interest and a superior alternative to the enzymatic method.

### 2.1. Enzymatic Electrochemical Biosensors

**Figure 2:**
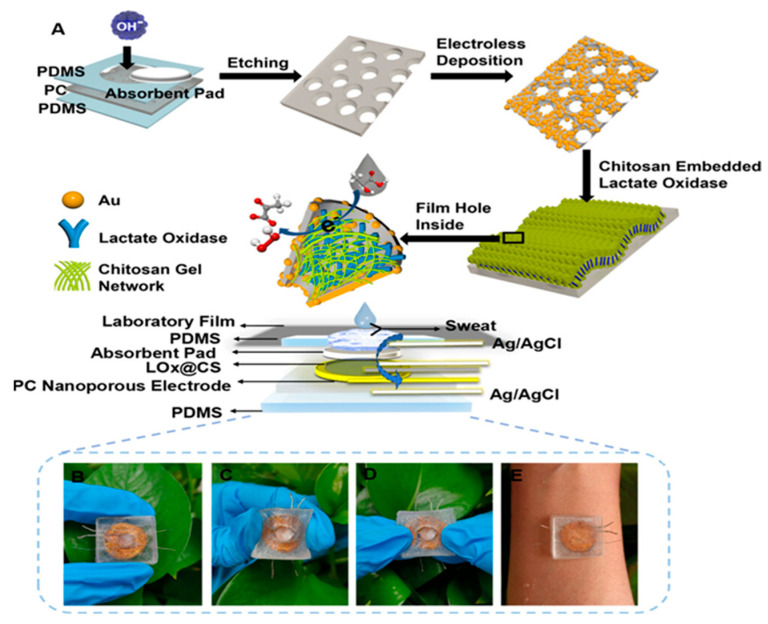
Schematic illustration of the preparation process of the stretchable LO*_x_*@CS PC sensor: (**A**) preparation of the LO*_x_*@CS PC sensor; (**B**–**E**) photograph of the prepared sensors for lactate detection in sweat. Reprinted with permission from [10]. Copyright © 2024 American Chemical Society.

The electrochemical detection method using lactate concentration in sweat is perhaps the most widely used approach in the existing literature. In this method, enzymes such as lactate oxidase (LOx) and lactate dehydrogenase (LDH) are commonly employed, and electronic mediators, such as Prussian blue [15], are used for effective electrochemical response. Lactate dehydrogenase is a very stable enzyme and can efficiently convert lactate into pyruvate with nicotinamide adenine dinucleotide (NAD) as a coenzyme. Kumar et al. [16] demonstrated that it is possible to detect lactate in sweat even in the absence of a redox probe, with a wide concentration range (0.1–100 mM). In this method, lactate dehydrogenase is immobilized on the surface of a working electrode using an EDC–NHS crosslinker (1-ethyl-3-(3-dimethylaminopropyl carbodiimide and *N*-hydroxysuccinimide). Prior to this, the SPCE is pretreated with oxygen plasma, followed by ECX electrochemical oxidation using 10% acrylic acid with a DC voltage of 1.8 V. In the case of LOx, lactate is recognized and treated enzymatically through an oxidation reaction in the presence of oxygen, generating pyruvate and hydrogen peroxide as products. The peroxide then undergoes a redox reaction induced by the electronic mediator, which leads to the passage of electrons inside the electrochemical cell, thus generating a detectable response. It should be noted that in these enzymatic methods, difficulties arise in obtaining linearized data. This is due to variations in external conditions such as pH and temperature, parameters that modify enzymatic activity and compromise its integrity. Enzyme-based sensors are more susceptible to pH changes, and it is possible to identify the underlying mechanisms by studying lactate oxidase activity under various reaction conditions. Importantly, the study by Ping Xu et al. [17] underlines that lactate oxidase achieves its maximum enzymatic activity in a relatively narrow pH range, with its peak activity at pH = 7.7. When moving towards acidic or basic pH, a rapid reduction in enzymatic activity occurs, thus compromising lactate detection in biological matrices. pH variations during lactate detection are associated with the redox reaction between the substrate and the electronic mediator, which generates either protons, thus acidifying the reaction environment, or hydroxyl ions, which have the opposite effect. For lactate detection, electrochemical biosensors can be combined with other types of sensors that provide important information on physical conditions such as temperature and pH. This also allows one to better control the measurement conditions of the metabolite, thus achieving its precise detection in sweat. In the study by Wu et al. [10], an electrochemical sensor was used to simultaneously measure the lactate concentration and the temperature by analyzing the transmembrane current and the current directly generated via the redox reaction. The sensor was composed of gold nanoelectrode arrays fabricated on the nanoporous polycarbonate (PC) membrane by encapsulating lactate oxidase (LOx) in chitosan (CS) hydrogel (Figure 2). Flexible gold nanoporous electrodes not only increased the electrode area but also provided a nanoconfined space to accelerate the catalytic reaction of LOx and control the substrate’s concentration on the surface of LOx to reduce substrate inhibition.

**Figure 3:**
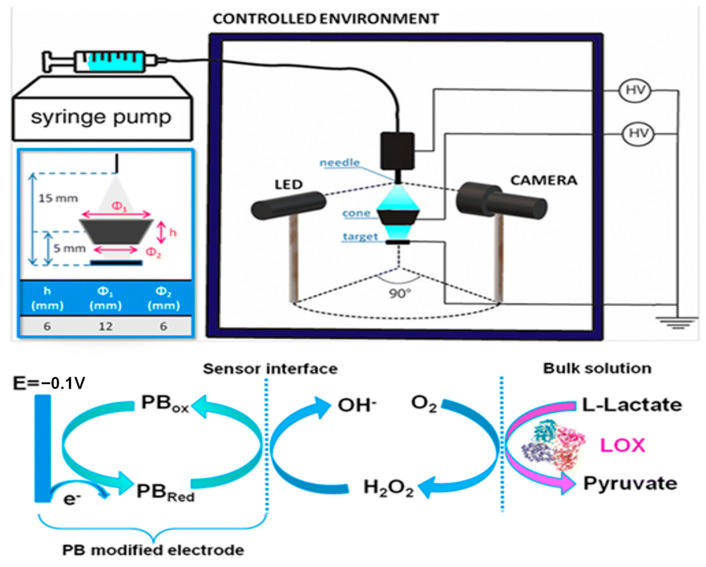
ESD setup (top right panel) and enlarged scheme of the deposition region (top left panel) where the dimensions and the relative distance between the needle of immobilization, the cone target, and the SPE are depicted. Schematic of the redox reactions that occur on the electrode surface during amperometric measurements (bottom panel). Reprinted with permission from [23]. Copyright 2024 Royal Society of Chemistry.

Prussian blue is not the only electronic mediator used in this field. Khan and Andreescu investigated a new material, called MXCeO2 (M represents an “early” transition metal and X represents carbon or/and nitrogen), which is obtained from the combination of cerium oxide with Mxenes and serves as a catalytic amplifier for electrochemical biosensors. They demonstrated that this substance undergoes an efficient catalytic action towards hydrogen peroxide reduction and is selective for oxidases (e.g., LOx) [18]. In another study, Xuan Weng [19] used a combination of nanozymes with peroxidase-like properties. The combination of cerium oxide with molybdenum sulfide led to an increase in active catalytic sites, resulting in enhanced catalytic efficiency. Following the redox reaction with hydrogen peroxide, a significantly intense electrochemical signal was obtained, which allowed for the accurate identification of lactate concentrations in sweat. In some methods, an electronic mediator may not be used. For instance, for the continuous monitoring of athletic performance, Shuling Deng used a working electrode made of oxidized graphene nanosheets and obtained a response with high sensitivity and a wide detection range, between 0.1 and 80 mM [20]. Enzyme immobilization using a working electrode is also very important since poor immobilization can lead to the inaccurate detection of the analyte. Thus, chemical compounds that enable optimal enzyme immobilization are often used. In Liang Tian’s study, lactate oxidase and peroxidase were anchored to the electrode via NiCo_2_O_4_ microspheres, coated with carbon nanotubes [21]. The microspheres were placed on the working electrode, thus improving its conductivity. Ferrocene–methanol has also been used as a redox mediator and is capable of lowering the reaction potential due to the effect of HRP. In this approach, the reaction byproducts are minimized due to the high redox potential of the process. A very recent study by Qingrong He et al. [22] also highlighted the possibility of achieving enzyme self-immobilization on the surface of the working electrode without the use of external crosslinkers. In this interesting study, the structure of lactate oxidase was modified through the modification of phenazine. Through this modification, they obtained accurate results for lactate detection in sweat, as phenazine has excellent electron transport properties, which reduce the detection potential for lactate. Enzyme immobilization is key in the successful development of enzyme-based biosensors. It must be highlighted that LOX is an unstable enzyme, and thus an appropriate immobilization technique is crucial for the fabrication of a robust biosensor. Several immobilization techniques have been investigated, among which a new technique using electrospray deposition at room temperature (ESD) (Figure 3) resulted in biosensors with superior performance. These devices have a high storage capability of up to 90 days and are not affected by low temperatures; moreover, they can be reused for up to 24 measurements on a new as well as a three-month-old electrode [23].

### 2.2. Enzyme-Free Electrochemical Biosensors—Semiconductors

**Figure 4:**
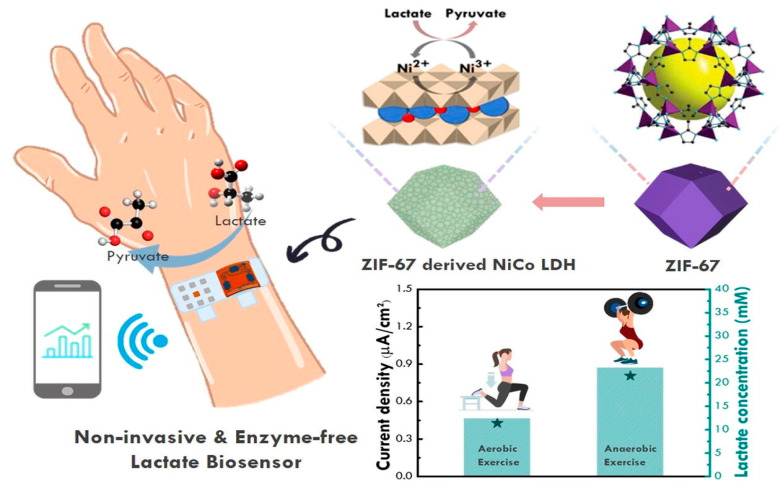
Schematic illustration of enzyme-free electrochemical biosensors, based on Ni derivatives. Ni in the oxidated state undergoes a redox reaction with lactate, resulting in its oxidation into pyruvate and the reduction of Ni to its +2 form. Reprinted with permission from [25]. Copyright 2024 Elsevier.

In these biosensors, enzymes are not used to detect lactate. Instead, this method involves the use of certain chemical–physical characteristics of inorganic compounds to “capture” the analyte through specific interactions. These biosensors have superior sensitivity for the detection of electrochemical signals; however, their poor selectivity remains the main unresolved challenge. The multiple analytes present in sweat can interfere with measurement; therefore, the detection methods using these biosensors are not as precise as those based on enzymes. Ling-Yu Chang [24] studied a typical non-enzymatic biosensor using a nickel–cobalt layered double hydroxide (LDH) capable of interacting with the lactate at its numerous electrochemical sites, thus inducing its oxidation. Furthermore, the biosensor involves the use of N-doped graphene quantum dots, which lower the resistive phenomena on the working electrode. The authors also demonstrated that the selective detection of lactate in a matrix such as sweat was still possible despite numerous interferences. To evaluate the selectivity of NiCo-based layered double hydroxides with N-doped graphene quantum dots (NGQD/m-NiCo LDH-based SPCE), an interference test was performed with a series of chemical compounds present in human sweat, such as glucose, ascorbic acid, and uric acid. The interference test results reveal a distinct response with lactate, whereas only minor fluctuations were observed after the addition of uric acid, ascorbic acid, and glucose. These results confirmed the superior selectivity of NGQD/m-NiCo LDH-based SPCE for lactate detection. Given its excellent electrochemical properties, nickel is widely used in non-enzymatic biosensors. Gao et al. employed nickel oxide nanoparticles placed on a graphene support. Nickel is present in its two oxidation states +2 and +3, and in NiOOH/Ni(OH)_2_, there is a crystalline core of metallic nickel inside a shell of NiO, with a crystalline/amorphous surface. Graphene was used to enhance the electrochemical sensitivity of the generated biosensor, which also yielded good results regarding linearity in lactate detection [14]. This device facilitates the accurate identification of electrochemical responses, with a wide detection range. Pei Li [12] developed a working electrode via the glancing angle deposition (GLAD) of nickel oxide on a gold surface. GLAD is a physical vapor deposition process that involves depositing a material onto a substrate oriented at oblique angles relative to a vapor source, typically accompanied by substrate rotation. This results in the formation of unique nanostructured thin films with columnar architectures, which increase the number of active sites available for catalysis, leading to a high surface-to-volume ratio. The increased surface area allows for a greater interaction between the analyte and catalyst, in which Ni in the oxidated state undergoes a redox reaction with lactate, resulting in the oxidation of lactate to pyruvate, while Ni is reduced to its +2 form (Figure 4), thus enhancing the sensitivity and lowering the detection limit of the sensor. Oxidized nickel holds promise as an alternative to lactate oxidase, yielding excellent results. It stands out among the different nanozymes (Co, Zn, and Cu) due its low toxicity, high catalytic activity, and economic feasibility.

### 2.3. MIPs Electrochemical Biosensors

**Figure 5:**
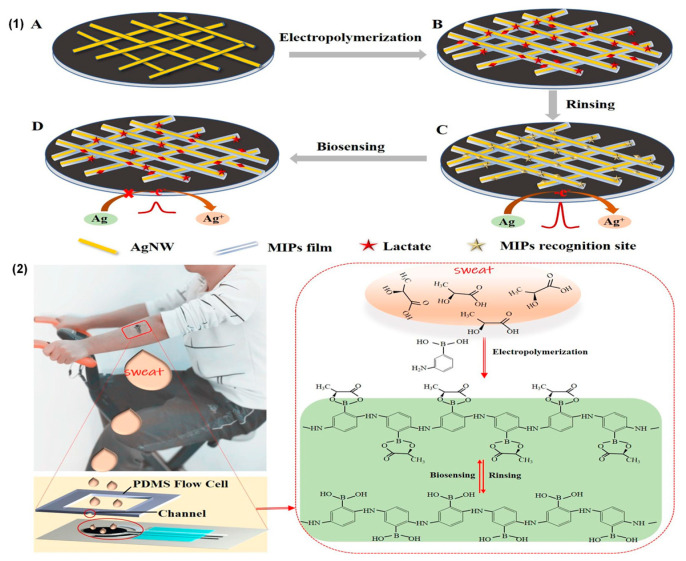
(**1**) The fabrication process of the MIP–AgNWs electrochemical biosensors for the epidermal monitoring of lactate; (**2**) the application of a screen-printed three-electrode biosensor chip on a male volunteer’s arm and the principles of MIP formation and biosensing. Reprinted with permission from [11]. Copyright 2020 Elsevier.

To date, the use of molecularly imprinted polymers seems to be a valid alternative to the use of the enzymatic approach. It is characterized by the direct electropolymerization of specific compounds onto the working electrode, together with a template molecule that must then be detected. Following the removal of the template (for example, through an overoxidation reaction), the polymer layer comprises highly specific areas for the target molecule, which are captured and subsequently detected through electrochemical reactions (Figure 5). This type of biosensor has very high selectivity and detection sensitivity; moreover, due to the absence of enzymes, the system is not affected by changes in pH and temperature. Additionally, electronic mediators can be used to increase their sensitivity in detecting electrochemical signals. In the study by Grace Dykstra [26], a working electrode was prepared using a silk-screened carbon electrode, with a deposited layer of Prussian blue which acted as the electronic transfer mediator. Subsequently, an MIP composed of aminophenylboronic acid and pyrrole molecules was developed via electropolymerization. In another recent study using MIPs with Prussian blue (PB) as an electronic mediator, lactate was used as the template molecule, and pyrrole (Py) served as the functional monomer. PB was embedded into the MIP as a built-in redox probe, thus eliminating the need for an additional probe and facilitating the simultaneous quantification of lactate concentration. Moreover, the MIP-doped platinum nanoparticles (PtNPs) enhanced the electron transfer ability, further improving the sensitivity of the sensors [27]. As demonstrated by Yangyang Chen [28], these biosensors can be easily integrated with the textile fabric, making the device suitable for wearing. They built a device with excellent electrochemical properties, which was implanted on a textile surface and could undergo many detection cycles.

## 3. Comparative Analysis of Non-Invasive Electrochemical Biosensors for the Detection of Lactate in Sweat

From a careful analysis of the biosensors included in this review, it can be concluded that enzyme-free devices have a very wide range of linear detection, due to the lack of any direct dependence on variations in pH and temperature during the analysis of lactate concentrations in sweat.

**Table 1:** Comparative analysis of non-invasive electrochemical biosensors for lactate detection in sweat.

Working Electrodes	Measurement Techniques	Typology	Sensitivity	Linearity	Detection Limits	Ref.
Nano–Au/CS PC	Amperometry	LOx enzyme	0.8 µA/mMcm^2^	0.01–35 mM	0.144 µM	[10]
Au/GLAD NiO	Amperometry	Enzyme-free	80 µA/mMcm^2^	1–65 mM	16 µM	[12]
NiOOH/Ni(OH)_2_	Amperometry	Enzyme-free	80 µA/mMcm^2^	0.02–53 mM	0.13 µM	[14]
SPCE/PB	Amperometry	LOx enzyme	−0.01 µA/mMcm^2^	1–20 mM	0.2 mM	[15]
SPCE	EIS	LDH enzyme	N/A	0.100 mM	0.1 mM	[16]
SPCE/MXCeO_2_	Amperometry	LOx enzyme	N/A	0.01–12 mM	0.4 mM	[18]
CeO_2_/MoS_2_AuNPs	Amperometry	LOx enzyme	0.027 µA/mMcm^2^	0–100 mM	N/A	[19]
GO	EIS	LOx enzyme	N/A	0.1–80 mM	N/A	[20]
NiCo_2_O_4_/SWCNTs	Amperometry	LOx enzyme	N/A	0–30 mM	39.9 µM	[21]
SPGE	Amperometry	LOx enzyme	25.58 µA/mMcm^2^	0–5 mM	0.135 mM	[22]
NGQD/NiCo LDH	Amperometry	Enzyme-free	62.63 µA/mMcm^2^	0–15 mM	0.3 mM	[24]
SPCE/PB/MIPs	DPV/EIS	MIPs	N/A	1–35 mM	0.62 mM	[26]
SPCE/PtNPs/Pt@MI	Amperometry	MIPs	N/A	0–1.5 mM	1.1 mM	[27]
MCF/PBPPY	Amperometry	MIPs	0.11 µA/mMcm^2^	0.01–25 mM	N/A	[28]
SPCE	Amperometry	LOx enzyme	0.9 µA/mMcm^2^	0.03–0.5 mM	N/A	[29]

The only enzymatic biosensor that is not affected by these variations is the device proposed by Xing Xuan et al. [15], which operates via a lipophilic membrane placed externally on the working electrode. This not only reduces the flow of sweat reaching the biosensor but also counteracts diffusion phenomena within the fluid, thus reducing the loss of linearity caused by pH changes. S. Deng obtained the best results in terms of detection range, but their study was carried out on simulated sweat and therefore does not fully consider real-world conditions, such as, for instance, the presence of interference [20]. This analysis also reveals that it is possible to replace enzymatic biosensors with MIPs, which exhibit superior selectivity and good linearity in the obtained data [26].

## 4. Blood and Sweat Lactate Analysis Using Electrochemical Biosensors

**Figure 6:**
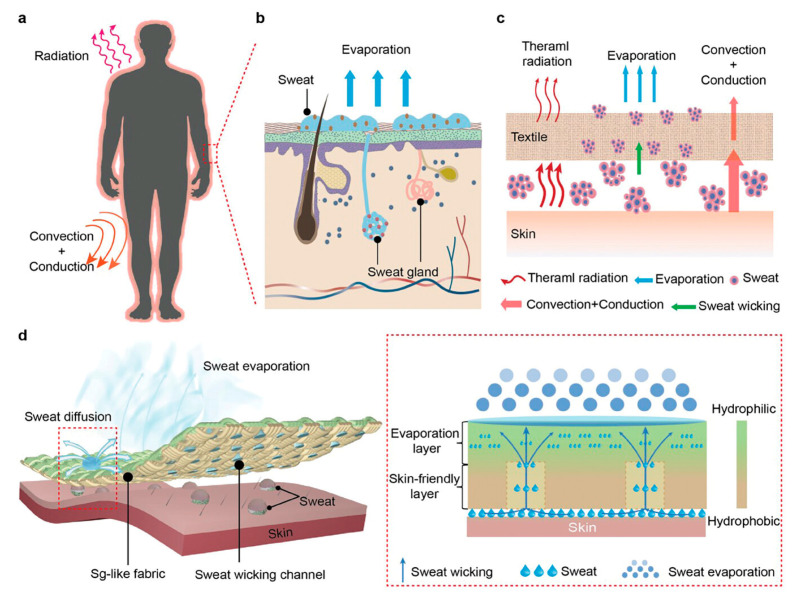
Schematic illustration of the complex mechanism of sweating from human skin with sudoriferous gland participation. (**a**) Illustration of heat transfer between the human body and the surrounding environment. (**b**) Sweat evaporation via sweat glands. (**c**) Schematic illustrating the heat transfer process from the human body covered with a textile to the surrounding environment. (**d**) Schematic illustration of the Sg-like fabric. Reprinted with permission from [13]. Copyright © 1999–2024 John Wiley & Sons, Inc., or related companies.

Sweating involves a very complex mechanism [13] (Figure 6), a phenomenon that can be identified through large lactate oscillations produced by the sweat glands. In fact, blood filtering is not involved in the passage of lactate into sweat, and its production is not linked to biochemical signals; rather, it is more related to the psychological, nervous, mechanical, and hormonal signals sent directly to the glands themselves [30].

Firstly, the correlation between the concentrations of lactate in blood and sweat is not yet clear. This is a major challenge since the data obtained from the detection of lactate in sweat could be significant for the analysis of the physiological state of an individual. A more in-depth review of the literature revealed that lactate detection in sweat is complex. Many factors should be considered in the development of a reliable biosensor. The direct generation of the metabolite by the sweat glands leads to large variations in the lactate concentration in sweat, which does not seem to be directly related to that of the blood [30]. The extent to which this phenomenon is dependent on the metabolism of the glands and how it can be predicted using mathematical algorithms remain unexplored. In a previous study, Garcia et al. developed a methodology for the accurate prediction of blood lactate values using a biosensor, which was able to reliably and accurately predict absolute blood lactate values, with only 0.3 mM of accumulated error compared to portable blood lactate meters, the current gold standard for sports clinicians [31].

**Figure 7:**
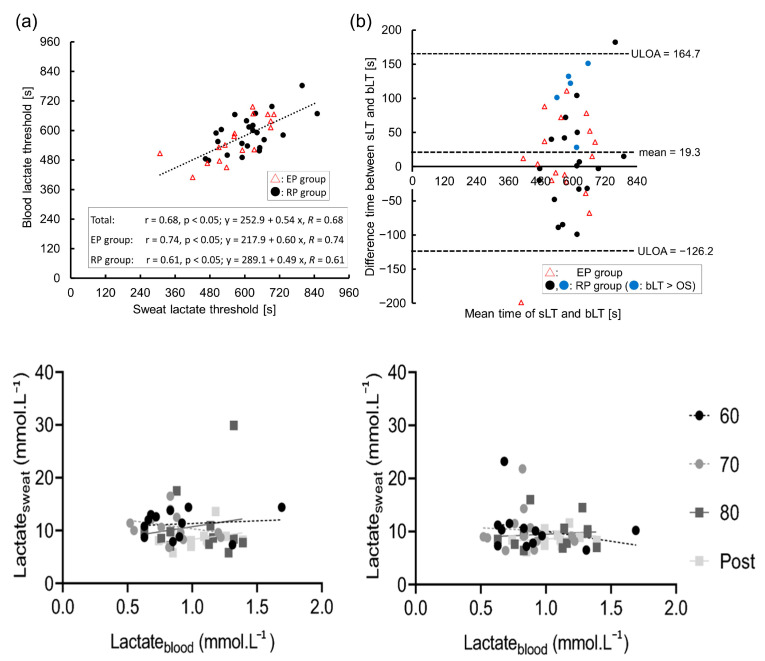
The relationship between the sweat and blood lactate thresholds (sLT and bLT): (**a**) scatter plot and approximation line between the sLT and bLT; (**b**) validity testing using Bland–Altman plots. The figure describes the correlations between lactate concentrations in blood plasma and upper arm sweat (left panels) and upper back sweat (right panels) during incremental cycling in the heat (33 °C, 65% relative humidity RH; n = 12). Black circles with a dashed regression line represent 60% maximum heart rate (HRmax (60)); grey circles with a dashed line represent 70% maximum heart rate (HRmax (70)); dark grey squares with a solid line represent 80% maximum heart rate (HRmax (80)); and light grey squares with a solid line represent post-exercise (post). Reprinted with permission from [30,34]. Springer Nature 2020 copyright. MDPI 2023 copyright.

Through targeted bibliographic studies, we explored this research gap in depth. Of the six articles analyzed, only two highlighted a correlation between lactate levels in blood and sweat. However, in analyzing the values obtained in these studies, we found significant differences. Sakharov [32] highlighted that lactate concentration is divided in capillary blood, venous blood, and sweat. The final correlation between lactate levels in venous blood and sweat is determined via equation C (lactate in venous blood) as 0.73 × C (lactate in sweat), which is quite different from the relation experimentally determined by Karpova [33] for the working muscle area and latent muscle area (angular coefficient = 5.7 and 2.6, respectively). As such, even if the correlation is mild or good (R^2^ > 0.95), there is no agreement between the different findings in the literature. This, therefore, suggests a degree of randomness in the values found when analyzing the two biological fluids, a hypothesis supported by the remaining four articles, in which the correlation is quite low, R^2^ < 0.9. This highlights that the correlation between the different concentrations of lactate in sweat is undetermined. In particular, Klous [30] and Maeda [34] revealed that lactate concentration in sweat does not change with variations in its concentration in the blood and vice versa (Figure 7).

Furthermore, studies should also focus on the dilution of lactate in sweat due to an increase in sweating, and the effect of variations in pH and temperature on this phenomenon, as well as understanding how it influences the detection of the analyte. In fact, it has been noted that all of these processes lead to a loss of linearity in biosensor responses used for lactate detection. Kudo sought to solve this problem by designing an “atypical” biosensor. To prevent measurement interference due to the reabsorption or evaporation of sweat, bacterial contamination, and pH variation, a wristwatch biosensor using PBS as a continuous carrier for sweat collection was designed [29]. Additionally, appropriate mathematical adjustments were made to correct measurements in real time, such that the integrity and reliability of the data could be preserved.

Moreover, to achieve improvements in biosensor responses, it is necessary to focus directly on the device parameters, among which the loss of linearity in biosensor responses considering the influences of pH, temperature, and the flow of sweat reaching the working electrode are the primary factors. Using a membrane that limits diffusion, placed directly above the working electrode, results in excellent biosensor responses at the expense of a reduction in sensitivity [15]. Nevertheless, this paves the path towards the application of enzymatic electrochemical biosensors. Furthermore, it is possible to integrate lactate oxidase with an inorganic compound, thus improving the immobilization of the enzyme on the electrode and enhancing the electrochemical response [21]. Another solution to reducing or eliminating the dependence of detection results on the pH and temperature conditions of the system could be to use different biosensors. The use of MIPs may avoid this problem, as they simply involve interactions between the polymer layer and the lactate itself. However, even in MIP applications, whether the generated interactions are affected by these variations remains unknown.

## 5. Conclusions

In future research, a more in-depth study of the physiological mechanism determining the presence of lactate in sweat is needed.

As can be seen from Table 2, there are still no biosensors in the market that can be used for lactate detection in sweat. The only exception is the IST device, but this is currently only capable of detecting levels in simulated sweat and is still under development. It uses a buffer to guarantee pH neutrality during the measurement; this achieves strong linearity in the obtained data and therefore success in lactate analysis.

**Table 2:** Current progress in the development of non-invasive devices for lactate sensing.

Company	Sample Type	Stage of Development	Measuring Range	Sample Volume	Testing Time	Operating Temperature
Zimmer & Peacock	Sweat	Research	N/A	N/A	N/A	N/A
Nova Niomedical	Blood	Market	0.5–25 mM	0.5 µL	10 s	10–45 °C
PKvitality	Sweat	Research	N/A	N/A	N/A	N/A
IST	Sweat	Market	0.05–25 mM	N/A	<90 s	15–42 °C
Cosmed	Blood	Market	0.5–25 mM	0.3 µL	15 s	N/A
APExBIO	Blood	Market	0.7–22.2 mM	3 µL	45 s	10–40 °C

**Figure 8:**
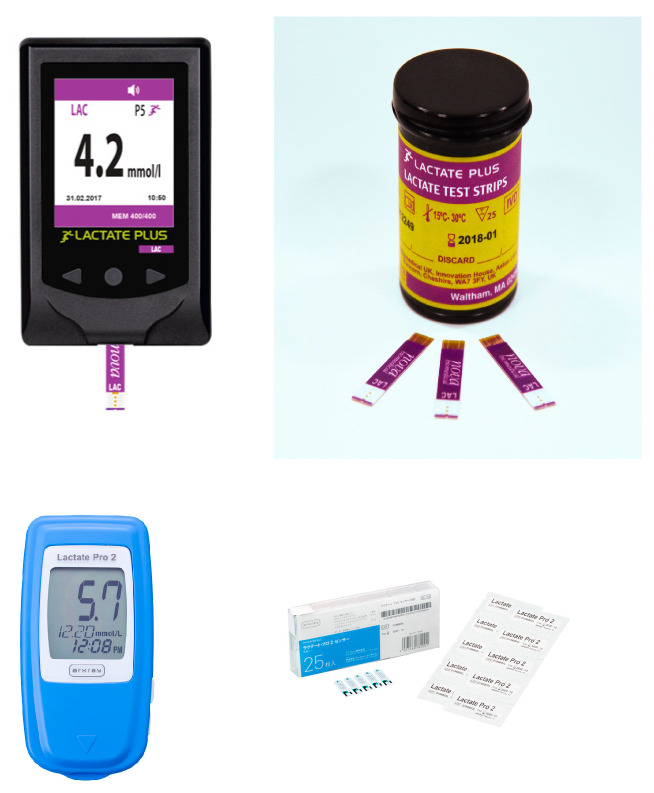
Examples of devices present on the market for lactate sensing. On the top, Lactate Plus is presented with lactate test strips, made by Nova Biomedical; below is Lactate Pro 2 with its strips made by Arkray. Reprinted with permission from Nova Biomedical Italy srl and from Arkray, Inc. © 2023 Nova Biomedical V1.128. Copyright© 2024 Arkray, Inc.

Currently, lactate detection in sweat may not yield the same results as those obtained with simulated sweat. The rest of the devices available on the market are non-wearable, invasive, and involve detection through blood analysis (Figure 8). The detection parameters of these devices are all very similar, and the lactate detection range reaches a maximum of 25 mM.

In conclusion, the development of biosensors with sensitivity and linear detection range compatible with sweat lactate concentrations seems more feasible than ever. The main problem remains the necessity of finding a biomedical correlation between lactate levels present in the blood with those present in sweat. For this purpose, a new European-supported project, called H2TRAIN, has been established to seek a definitive solution to the problems listed in this minireview. H2TRAIN employs a new cutting-edge biosensor for the detection of various parameters in sweat, including lactate, cortisol, and C-reactive protein. These detectors are based on graphene oxide, a 2D material that is functionalized as a detection biomarker, complemented by edge-cloud AI. The edge-cloud AI continuum is a novel concept, and this project will endow biosensors with smart properties for monitoring the levels of different compounds in sweat [35]. The project encompasses the entire supply chain, from R&D and the manufacturing of microsystems to electronics assembly, as well as physical integration in wearables and textiles and the integration of smart systems (implementation of software and AI algorithms). The key issues raised by this technology will be addressed. The design, testing, and validation of the proposed solutions will be carried out in medical applications, as well as from the perspective of sports sciences, with a substantial contribution from the social sciences and humanities. Guidance offered by end-user cohorts and external organizations related to different stages of the development cycle will help in advancing future applications of the technology [36]. The problem of finding a correlation between lactate levels present in the blood with those present in sweat could be addressed, first and foremost, through artificial intelligence algorithms [personal communication by Prof. Juan A. Montiel-Nelson, University of Las Palmas, Spain]. In fact, data on lactate concentrations, collected through measurements using blood as a matrix, could be analyzed and compared with data related to the oscillations in lactate concentrations in sweat. Through in-depth analysis and post-measurement correction, it would perhaps be possible to fill the research gap that continues to exist regarding the correlation between lactate concentrations in the two biological matrices. Finally, lactate concentrations in sweat could be used to depict patients’ pathological conditions.

## Data Availability

As this is a review article, no data were generated.
